# Effects of Dexmedetomidine and Midazolam on Motor Coordination and Analgesia: A Comparative Analysis^☆^

**DOI:** 10.1016/j.curtheres.2013.05.003

**Published:** 2013-12

**Authors:** Mustafa Said Aydogan, Hakan Parlakpinar, Mehmet Ali Erdogan, Aytac Yucel, Muharrem Ucar, Mustafa Sağır, Cemil Colak

**Affiliations:** 1 Department of Anesthesiology and Reanimation, Inonu University, Faculty of Medicine, Malatya, Turkey; 2 Department of Pharmacology, Inonu University, Faculty of Medicine, Malatya, Turkey; 3 Department of Biostatistics and Medical Informatics, Inonu University, Faculty of Medicine, Malatya, Turkey

**Keywords:** accelerod, dexmedetomidine, hot plate, midazolam, motor impairment, rotarod, tail flick

## Abstract

**Objective:**

We compared the effects of 2 sedative drugs, dexmedetomidine and midazolam, on motor performance and analgesic efficacy in a rat model.

**Materials and methods:**

Rats were randomly divided into the following 4 groups on the basis of the treatment received. The first group received 83 µg/kg/min midazolam; the second, 1 µg/kg/min dexmedetomidine; the third, 83 µg/kg/min morphine; and the fourth was a control group. The rats were measured motor coordination and pain reflexes by using rotarod, accelerod, hot plate, and tail flick tests.

**Results:**

At all the tested speeds, the midazolam-injected rats remained on the rotarod longer than did the dexmedetomidine-injected rats. Furthermore, in the 10-minute accelerod test, the midazolam-injected rats remained for a longer duration than did the dexmedetomidine-injected rats. The latency time for the hot plate test was significantly higher at 10 minutes and 20 minutes in the dexmedetomidine group than in the midazolam group. Further, the latency time at 10 minutes for the tail flick test was greater in the dexmedetomidine group than in the midazolam group.

**Conclusions:**

In this rat model, midazolam results in faster recovery of motor coordination performance when compared with dexmedetomidine.

## Introduction

Dexmedetomidine and midazolam are known to be excellent drugs for sedation/anesthesia. Dexmedetomidine is a highly selective α_2_-adrenergic agonist; it is both a sedative and an analgesic agent.^1^ Midazolam is another commonly used intravenous sedative agent; midazolam’s metabolites have relatively long half-lives, particularly after repeated administration, its use may lead to prolonged sedation and induce hangover effects such as cognitive and psychomotor impairment.^2,3^

To our knowledge, no study on comparative motor performance and analgesic efficacy has satisfactorily investigated the effects of these 2 drugs. Therefore, in this study, we aimed to compare the sedative and analgesic effects and the recovery profiles of midazolam and dexmedetomidine in a rat model by conducting motor coordination tests (rotarod-accelerod test) and by evaluating the analgesic response times by conducting hot plate and tail flick tests.

## Methods

This study was approved by the Inonu University Research Animals Ethics Committee of Inonu University, Malatya, Turkey (Acceptance No. 2012/A-55).

### Animals and laboratory

This animal experiment was conducted in accordance with EC Directive 86/609/EEC. Thirty-two male Sprague-Dawley albino rats (age 10–12 weeks; weight, 200–260 g) were obtained from the Inonu University Laboratory Animal Research Center and placed in a temperature- and humidity-controlled room (21°C [±2°C]; 60% [5%] humidity) with a 12-hour light:dark cycle. Food and water were provided ad libitum, except during the test periods. The rats were randomly divided into 4 groups (n = 8 per group) on the basis of the treatment received: 0.9% sodium chloride saline (control), morphine hydrogen chloride⁎ midazolam†, and dexmedetomidine‡ .

### Drug application

The rats were anesthetized by IP administration of 100 mg/kg ketamine§ and 10 mg/kg xylazine║. The right jugular vein was catheterized for drug and vehicle administration. Morphine hydrochloride, midazolam, and dexmedetomidine were administered in a saline solution as 1 mL. Morphine hydrochloride and midazolam were administered at 83 µg/kg/min and dexmedetomidine was given at 1 µg/kg/min. The dosage scheme was chosen according to that used in previously reported related successful studies.^4–6^ There was sufficient time between the anesthesia and testing of the test drugs. However, this washout period was more different among the rats. For standardization of the experimental design, first of all the zero point was determined according to the sensory-motor responses as described previously.^7^

Sensory processing was evaluated in paw withdrawal response to forceps pinch of the lateral foot/toe. The pinch was limited to a maximum of 1 second to avoid direct paw tissue trauma. Sensory responses were evaluated by the withdrawal reflex or vocalization to pinch and quantified as 0. Motor function was quantified as 0 = normal dorsiflexion ability and normal walking without curled toes.

### Experimental procedures in the rotarod and accelerod tests

Rotamex 4/8 system (Columbus Instruments, Columbus, Ohio) was used for the rotarod and accelerod tests. Trials were carried out to select the test group 24 hours before the experiment. The rats were placed on a rotating rod, and the test was performed at different rotation speeds (for a maximum of 5 minutes per speed level), beginning at the slowest speed of 5 rpm, followed by 10 rpm, and then with 10-rpm increments in the speed up to 40 rpm, while measuring the duration for which each animal could stay on the rod at a given rotation speed. Animals that could remain on the rod for 2 successive trials were selected for drug testing. Results were expressed as the percentage of animals that succeeded in remaining on the rod until the cut-off time (300 seconds) was reached. The total time that the rats ran on the rotarod, the time to falling from it, and all other set-up parameters were recorded. The accelerod test was set up with acceleration from 1 to 79 rpm within 4 and 10 minutes. Because several rats were tested in a single session, each rat was allowed to rest for approximately 5 minutes between the different speed tests; this helped to reduce the animal’s stress and fatigue.^8^

### Measurement of analgesia

Acute thermal pain was modeled by the hot plate and tail flick tests, which are 2 methods to measure thermal analgesia in rodents.

### Experimental procedures of the hot plate test

The surface of a hot plate (Columbus Instruments) was heated to achieve a constant temperature of 50°C (±0.5°C), which was ascertained using a built-in digital thermometer. The time (in seconds) between the placement of the rats on the plate and the onset of shaking, paw licking, and jumping off the plate was recorded as the response latency. To avoid tissue damage, 60 seconds was set as the cut-off time after which the rats were returned to the cage, regardless of whether or not a response was observed.^9^ The baseline was considered to be the mean reaction times obtained at 0 and 30 minutes before administration of the drugs and was defined as the normal reaction of the animal to temperature stimulus. Latency in the hot plate test was measured at 0, 10, 20, and 60 minutes after drug injection. No further nociceptive thresholds were measured until full recovery occurred from the effects of the general anesthetic.

### Experimental procedures in the tail flick test

Antinociception and thermal analgesia were assessed using the radiant heat tail flick test apparatus (Type 812; Columbus Instruments) as previously described.^10^ Briefly, the rats were placed in transparent hard plastic tubes, and the tests were repeated 3 times (with a 15-second interval) at each time point. The mean tail flick latency obtained from measurements of 3 pre-drug trials represented the individual baseline latency. Only those animals showing tail flick latencies ranging from 2 to 5 seconds before the treatment were used in the experiments. Immediately after baseline assessment, drugs or saline was injected into the rats according to the study protocol. Measurement of responses were taken at time zero, 10, 20, and 60 minutes after treatment of the animals with drugs by application of pressure from the analgesiometer onto their tail (1 cm from the tip of the tail). The cut-off time was set at 10 seconds to avoid tissue damage. The timing of the drug injections was adjusted according to those used in previously reported related successful studies in rodents.^11,12^

### Statistical analysis

For detecting even minor effects, the required sample sizes used in this experiment were identified using statistical power analysis. The sample sizes necessary for a power of 0.80 were estimated using NCSS software (NCSS LLC, Kaysville, Utah). Data were analyzed using the SPSS software program for Windows, version 21.0 (IBM Corp, Armonk, New York). The assumption of normal distribution was confirmed using the Kolmogorov-Smirnov test. Homogeneity of variance was tested by Levene's statistic. When the assumptions of a normal distribution and homogeneity of variance were provided, 1-way analysis of variance was used to compare the investigated variables among the groups. Multiple comparisons were made by Tukey's honestly significant difference test when the variance was homogeneous; otherwise, Tamhane’s T2 test was performed for multiple comparisons. The results are expressed as mean [SD] for hot plate and tail flick tests, jugular catheter time, drug onset time, and recording onset time. The Kruskal-Wallis H test was used when the assumption of normality was not provided. The Mann Whitney *U* test with Bonferroni correction was used for multiple comparisons. The values were given as median (minimum–maximum) for rotarod and accelerod results, ketamine dose, and xylazine dose. Statistical significance was set at *P* < 0.05.

## Results

### *Catheterization and drug measurements*

The hypnotic effects of dexmedetomidine, midazolam, and morphine were observed within 30 seconds, and none of the rats moved spontaneously while the 3 drugs were being infused. As expected, there was no difference for the ketamine and xylazine doses among the groups (*P* > 0.05) (Table I). Drug onset and jugular catheter application times did not differ significantly among the groups (*P* > 0.05) (Table I). The onset time was found to be higher in the midazolam-treated group than in the dexmedetomidine group (*P* < 0.05) (Table I).

### Measurement of sedation

There was no difference among the groups in the basal rotarod performance measurements (*P* > 0.05) (Table II). A difference was observed only at 30 rpm between the midazolam and control group during the test (*P* < 0.016) (Table II). In the rotarod test, the duration of time for which each animal was able to stay on the rod, was found to be higher in the midazolam-treated group than in the dexmedetomidine group at all tested speeds (*P* < 0.016) (Table II). The dexmedetomidine and control groups showed a significant difference at all measurement points during the rotarod test (*P* < 0.016) (Table II). The duration of time for which the animals were able to remain on the rod was lower in the dexmedetomidine group than in the control group at all tested speeds in the rotarod test (*P* < 0.016) (Table II). For accelerod performance, there was no significant difference among the groups in the basal performance measurements (*P* > 0.05) (Table II). After the drug treatment, the duration for which each animal could stay on the rod during the accelerod test was significantly increased in the midazolam group than in the dexmedetomidine group at the 10-minute test (*P* < 0.016) (Table II). During the accelerod test, differences were observed between the dexmedetomidine group and control group for all measurements (*P* < 0.016) (Table II), whereas between the midazolam and control groups, differences were observed only at 4 minutes (*P* < 0.016) (Table II).

### Measurement of analgesia

Morphine showed longer latency times when compared with midazolam, dexmedetomidine, and control groups in the hot plate test (*P* < 0.016) (Table III). Compared with the control group, the midazolam group showed significantly increased latency times at 0, 10, 20, and 60 minutes in the hot plate test (*P* < 0.016) (Table III). At 10 minutes, the latency time of the dexmedetomidine group was longer than that of the midazolam group (*P* < 0.016) (Table III). The results of the hot plate test obtained at 10 minutes showed that the analgesic effect of dexmedetomidine (relative to the control group) was higher than that of midazolam (*P* < 0.05) (Table III). Compared with morphine, the midazolam, dexmedetomidine, and control groups showed more than decreased latency times at time points in the tail flick test (*P* < 0.05) (Table IV). Compared with the control group, the midazolam and dexmedetomidine groups showed significantly increased latency times at 0, 10, 20, and 60 minutes in the tail flick test (*P* < 0.05) (Table IV). At 0, 10, and 20 minutes, the latency times of the dexmedetomidine group were longer than those of the midazolam group (*P* < 0.016) (Table IV). The results of the tail flick test obtained at 10 and 20 minutes showed that the analgesic effect of dexmedetomidine (relative to the control group) was better than that of midazolam (*P* < 0.05) (Table IV).

## Discussion

The main finding of our study is that although midazolam exerted a faster onset recovery of motor coordination performance, dexmedetomidine provided greater analgesic efficacy and longer motor and sensory blockades than midazolam. This result is in agreement with a human-subjects study in which dexmedetomidine was reported to have a longer recovery time than a midazolam when used for sedation.^13^

Dexmedetomidine is the active isomer of the analgesic medetomidine that binds to α-2-adrenergic receptors in an agonist fashion with high specificity. The α-2-adrenergic receptor agonists produce varying levels of sedation, analgesia, muscle relaxation, and anxiolysis.^14^ Benzodiazepines, when administered alone, have been shown to have a hyperalgesic effect.^15^ Further, subcutaneous injection of midazolam decreased the analgesia associated with ketamine.^16^ Our findings emphasized the significantly greater analgesic status conferred by dexmedetomidine than by midazolam. It is not surprising to find that dexmedetomidine provided a better analgesic effect than midazolam. Boehm et al^17^ found that midazolam had no significant effect on the tail flick latency when compared with baseline. In contrast, dexmedetomidine showed a clear dose-dependent increase in tail flick latency. These findings are supported by the fact that patients treated with dexmedetomidine can report their pain response to health care providers as a result of their more conscious state.^18^

The hot plate test is commonly used to assess narcotic analgesia. Although the central and peripheral analgesics respond by inhibiting the number of contractions provoked by chemical pain stimuli, only the central analgesics increase the time of response in the hot plate test, because the hot plate is a specific central antinociceptive test in which opioid agents exert their analgesic effects via supraspinal and spinal receptors.^19^ In the hot plate test (Table III), treatment with morphine caused a marked increase in the latency time of the animals compared with other study drugs. It is because morphine shows antinociceptive efficacy through opioid receptor-mediated mechanisms achieved at doses that had no significant effect on motor activity. The results of the hot plate test clearly indicated that dexmedetomidine exerted its analgesic effect early (within the first 10 minutes). Furthermore, the results of the tail flick test obtained at 10 and 20 minutes showed that the analgesic effect of dexmedetomidine (relative to the control group) was better than that of midazolam. Therefore, the long-term analgesic effects of dexmedetomidine could be clinically useful for sedation. However, the recovery time from motor coordination impairment was more rapid in the midazolam group than in the dexmedetomidine group. Thus, midazolam may allow neurologic assessments and communication with the patient without interruption of the calming effects of sedation. The increased analgesic requirement in the midazolam group can be considered to be a normal response of the physiologic and compensatory mechanisms. This is consistent with characteristics of dexmedetomidine, which include the ability to achieve sedation while preserving patient arousability.^20^

Another possible explanation for this result may be related to the dexmedetomidine dose used in this study: Only a single concentration was evaluated. This may also be considered a limitation of our study.

## Conclusions

Taken together, these results indicate that midazolam is preferred over dexmedetomidine for use in sedation applications for recovery of motor coordination performance. Further studies are needed to evaluate the potential of these drugs for analgesia with psychomotor performance function.

## Conflicts of Interest

The authors have indicated that they have no conflicts of interest regarding the content of this article.

## Figures and Tables

**Table I:** Initial anesthesia dose and pretest time. Values are presented as median (minimum-maximum) or mean [SD].

Variable	Midazolam (n = 8)	Dexmedetomidine (n = 8)	Morphine (n = 8)	Control (n = 8)
Ketamine dose (mg)	30 (30–33)	30 (30–32)	30 (30–33)	30 (30–33)
Xylazine dose (mg)	4 (3–5)	4 (3–5)	4 (4–4)	4 (3–5)
Jugular catheter time (min)	14.50 [0.92]	14.25 [1.28]	14 [1.06]	14.12 [1.24]
Drug onset time (min)	26.87 [2.29]	26.75 [2.12]	27.25 [2.37]	27.50 [2.50]
Recording onset time (min)	148.75 [13.15]*^,^†	123.25 [9.16]*	112.37 [9.08]*	58.75 [16.48]
Wash-Out time (min)	198.57 [17.09]*^,^†	170.25 [12.10]*	150.30 [15.24]*	100.75 [19.21]

⁎ Versus control (*P* < 0.05).

† Versus morphine (*P* < 0.05).

**Table II:** Rotarod and accelerod results with or without (baseline) drugs. The data are presented as median (minimum–maximum).

Variable	Midazolam (n = 8)	Dexmedetomidine (n = 8)	Control (n = 8)
Baseline rotarod test (sec)			
5 rpm	300 (247–300)	300 (288–300)	300 (149–300)
10 rpm	300 (136–300)	300 (148–300)	255 (131–300)
20 rpm	180 (90–300)	175 (75–300)	184 (94–300)
30 rpm	45 (28–156)	39 (15–129)	45 (17–142)
40 rpm	17 (10–43)	15 (10–35)	18 (10–37)
Rotarod test with drugs (sec)			
5 rpm	224 (145–300)†	141 (110–160)*	300 (187–300)
10 rpm	198 (68–300)†	109 (99–126)*	300 (189–300)
20 rpm	144 (98–300)*^,^†	58 (20–117)*	215 (91–300)
30 rpm	38 (23–179)*^,^†	10 (10–20)*	191 (71–240)
40 rpm	14 (10–57)†	9 (6–16)*	45 (10–102)
Baseline Accelerod test (sec)			
4 (min)	91 (21–219)	137 (31–203)	136 (96–195)
10 (min)	142 (58–300)	184 (57–266)	225 (167–277)
Accelerod test with drugs (sec)			
4 (min)	102 (92–125)*	59 (13–94)*	154 (111–198)
10 (min)	163 (136–359)†	129 (113–136)*	223 (79–390)

⁎ Versus control (*P* < 0.016).

† Versus dexmedetomidine ( *P* < 0.016).

**Table III:** Hot plate latency time results (in seconds). Data are presented as mean [SD].

Variable (min)	Midazolam (n = 8)	Dexmetedomine (n = 8)	Morphine (n = 8)	Control (n = 8)
Baseline 0	20.6 [4.1]	19.1 [5.1]	19.5 [3.2]	18.5 [2.8]
Drug 0	22.5 [2.6]*^,^‡	23,1 [1.3]*^,^‡	36 [3.6]‡	14 [5.6]
Drug 10	17.6 [1.9]*^,^‡	18.6 [4.3]*^,^†^,^‡	34.8 [4]‡	13.8 [4.9]
Drug 20	15 [3.7]*^,^‡	14.8 [4.2]*^,^‡	24.7 [4.2]‡	12.6 [5.2]
Drug 60	11.8 [3]*^,^‡	11.5 [4.7]*^,^‡	17.1 [4.5]‡	11.1 [5.1]

⁎ Versus morphine.

† Versus midazolam.

‡ Versus control (*P* < 0.016).

**Table IV:** Tail flick latency time results (in seconds). Data are presented as mean [SD].

Variable (min)	Midazolam (n = 8)	Dexmetedomine (n = 8)	Morphine (n = 8)	Control (n = 8)
Baseline 0	3.31 [1]	3.54 [1.1]	2.77 [1.1]	3.42 [0.9]
Drug 0	8.95 [2.7]*^,^‡	9 [3.1]*^,^‡	12 [2]*^,^‡	3.76 [0.7]
Drug 10	8.50 [1.3]*^,^‡	8.75 [2.9]*^,^†^,^‡	11 [2]*^,^‡	3.85 [0.6]
Drug 20	6.88 [1.8]*^,^‡	7.62 [2.3]*^,^†^,^‡	9.87 [1.9]*^,^‡	3.86 [0.6]
Drug 60	6.1 [2.1]*^,^‡	5.75 [1.9]*^,^‡	8 [2]*^,^‡	3.75 [0.6]

⁎ Versus morphine.

† Versus midazolam.

‡ Versus control (*P* < 0.05).

## Footnotes

^⁎^ Trademark: Morphine^®^ (Galen, Istanbul, Turkey).

^†^ Trademark: Dormicum^®^ (Dem, Melsungen, Germany).

^‡^ Trademark: Precedex^®^ (Hospira, Lake Forest, Illinois).

^§^ Trademark: Ketalar^®^ (Pfizer, Istanbul, Turkey).

^║^ Trademark: Rompun^®^ (Bayer, Toronto, Ontario, Canada).
